# Homeodomain-interacting protein kinase 2 (HIPK2): a promising target for anti-cancer therapies

**DOI:** 10.18632/oncotarget.14723

**Published:** 2017-01-18

**Authors:** Yuanyuan Feng, Lihong Zhou, Xiaoting Sun, Qi Li

**Affiliations:** ^1^ Department of Medical Oncology, Shuguang Hospital, Shanghai University of Traditional Chinese Medicine, Shanghai, China

**Keywords:** HIPK2, cancer, p53, apoptosis, angiogenesis

## Abstract

The HIPK2 (serine/threonine homeodomain-interacting protein kinase 2) is a “caretaker” gene, its inactivation increases tumorigenicity while its activation inhibits tumor growth. This report reviews the anti-tumorigenic mechanisms of HIPK2, which include promotion of apoptosis, inhibition of angiogenesis in hypoxia, prevention of tumor invasion/metastasis and attenuation of multidrug resistance in cancer. Additionally, we summarize conditions or factors that may increase HIPK2 activity.

## INTRODUCTION

Since being discovered in 1998 by Kim et al. using a yeast two-hybrid screen designed to characterize novel molecules that bind to homeoproteins, the homeodomain-interacting protein kinases (HIPKs), have been proven to be a tumor suppressor and one of the highly conserved factors regulating signaling and gene expressions. They control a wide spectrum of biological functions such as DNA damage response, apoptosis, hypoxia, cell proliferation and invasion [1, 2, 3, 4].

The HIPK family members 1 to 3 are structurally similar, with HIPK4 being remotely related to them. HIPK1 to 3 were originally characterized as molecules that interact with, homeobox transcription factors NKx-1.2. Approximately 90% of the amino acid sequences that make up their kinase domains are conserved across HIPK1-3. Additionally, the architecture of their noncatalytic regions is also conserved [5, 6]. HIPK2 inhibits tumor growth through multiple mechanisms: promoting apoptosis, inhibiting angiogenesis, tumor invasion and metastasis by regulating various genes and signaling molecules such as p53 [7], JNK [8], Wnt [9], and VEGF [10]. Conditions and factors that lead to HIPK2 activation, such as ionizing radiation [11], ultraviolet light [12] and zinc [13], attenuate caspase-mediated cleavage of auto-inhibitory domain and ubiquitination, resulting in simulation, acetylation as well as phosphorylation of the protein [2, 14]. Scientific consensus suggests, HIPK2 is a “caretaker” gene: its inactivation increases tumorigenicity [15] and its activation inhibits tumor growth [6]. In this review, we discuss the function of HIPK2 and factors that may increase HIPK2 activity in order to expand current understanding of its anti-tumor effects.

## HIPK2 STRUCTURE

**Figure 1:**
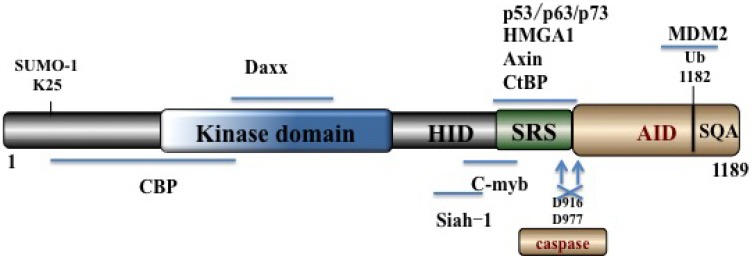
Schematic summary of HIPK2 domain structure It contains an N-terminal kinase domain, sumoylation site, kinase domain, HID, SRS and a C-terminal auto-inhibitory domain (AID) with the K1182 ubiquitination site.

HIPK2, a nuclear body localized 1189-amino-acid protein, belongs to the DYRK serine/threonine homeodomain-interacting kinase family [16]. HIPK2 contains an N-terminal kinase domain, a SUMO (small ubiquitin-related modifier) attachment site [17], a protein-protein interaction region, a homeobox-interacting domain (HID), a speckle-retention signal (SRS) domain [16, 18] (required for the subcellular localization of HIPK2 to nuclear bodies), and a C-terminus abundant in repeats of serines, glutamines and alanines (SQA region, also called tyrosine/histidine (YH)-rich region) [16, 18]. The C-terminus also contains an auto-inhibitory domain (AID) with a K1182 ubiquitination site which is ubiquitylated by p53 inhibitor mouse double minute 2 (MDM2) [20] (Figure 1). When cleaved by caspases on D916 and D977, full activation of HIPK2 ensues [19, 21].

## FUNCTIONS OF HIPK2

### HIPK2 promotes cancer cell apoptosis

It has been reported that HIPK2 is a tumor-inhibiting factor and DNA damage monitoring kinase by promoting apoptosis through targeting p53 and its family members, p73 and p63 [22], anti-apoptotic trans-repressor C-terminal binding protein (CtBP) [23, 24], MDM2 [25], Caspase-dependent processing [14] and the scaffold protein Axin [26]. In addition, factors associated with the pro-apoptotic effects of HIPK2, such as high mobility group AT-hook 1 (HMGA1) and Non-receptor tyrosine kinase (Src) overexpression, maintain the nuclear localization of HIPK2 and cellular sensitivity to apoptosis [26–28]. Human Papillomavirus E6 Proteins (HPV23 E6) inhibits HIPK2-mediated phosphorylation of p53 at Ser46 by disrupting HIPK2/p53 complex [29]. These lines of evidence demonstrate that the promoting role of HIPK2 in apoptosis involves various signaling pathways.

**Figure 2:**
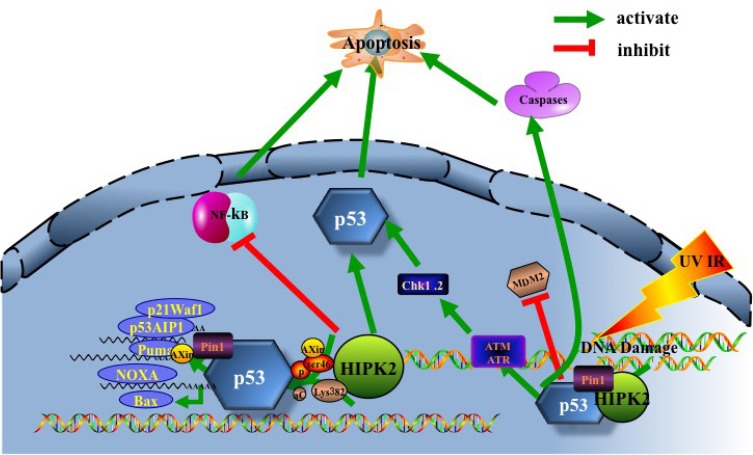
HIPK2/p53 induce apoptosis UV, IR-induced DNA damage facilitates activation of the DNA damage-activated protein kinases ATM and ATR. ATR and ATM in turn phosphorylate/activate downstream checkpoint kinases Chk1, Chk2, and tumour suppressor p53. Furthermore, ATM and ATR mediate HIPK2 activation by facilitating its stabilization through phosphorylation of the HIPK2 ubiquitin ligase. HIPK2 contributes to p53 apoptotic activation by inducing Ser46 phosphorylation, lysine 382 (Lys382) acetylation and Axin. Pin1 can induce HIPK2 stabilization and p53 Ser46 phosphorylation, essential for induction of apoptosis. HIPK2-mediated phosphorylation of p53 at Ser46 potentiates the activation of pro-apoptotic p53 target genes such as p53AIP1, p21Waf1, Noxa, Bax and Puma, resulting in cell death. In addition, HIPK2 can induce apoptosis through direct caspase activation and NF-kB pathway inactivation.

The key step for HIPK2 to promote apoptosis is phosphorylating and activating p53 at serine 46 [7, 31, 32]. p53 is a widely studied transcription factor that tightly regulates cellular responses to stress signals, *via* modulating expression of certain genes that impact growth arrest, senescence, apoptosis and DNA repair [33]. It can be inactivated by genetic mutations or deregulated by regulatory proteins [34] and other mechanisms, such as the up-regulation of its E3 ubiquitin ligase MDM2 [33]. HIPK2 is also among the kinases, such as DNA damage checkpoint kinases Ataxia telangiectasia mutated (ATM), ATM and Rad3-related (ATR) as well as their downstream checkpoint effector kinases 1 (Chk1), checkpoint effector kinases 2(Chk2), that regulate p53 protein stability, activity and target gene specificity, [34, 35]. HIPK2 induces p53-dependent apoptosis through phosphorylating p53 at Ser46 and acetylating p53 at Lys382 [12, 36] (Figure 2). MDM2-dependent HIPK2 degradation blocks p53 activation to promote cell survival. Apoptosis can be initiated by repressing HIPK2 degradation, strongly suggesting that HIPK2 is a potential target for cancer therapy [37, 38]. Upon sensing DNA damage, HIPK2 interacts with itself and undergoes autophosphorylation at Thr880/Ser882 [39]. The prolyl isomerase (Pin1) serves as a necessary auxiliary factor for stabilizing HIPK2 following DNA damage, hence essential for apoptosis induction [39, 40]. Axin mediates interaction between Death domain- associated protein 6 (Daxx) and p53. When DNA damage occurs due to UV irradiation, the nucleus-translocated Axin interacts with Daxx and two forms of p53 (one bound with Axin, and the other with HIPK2). In the Axin-Daxx-p53-HIPK2 complex, activated HIPK2 induces phosphorylation of p53 at Ser46, which is further enhanced by Axin and Daxx. Axin-activated p53 induces apoptosis through transactivating target genes such as p53 up-regulated modulator of apoptosis (PUMA) [41]. Of note, promoting cancer cell apoptosis is the main rationale behind the cancer treatment strategies that utilize ionizing radiation and chemotherapeutic drugs. HIPK2 interacts with the C-terminus of p53 to phosphorylate the NH2-terminal Ser46 to initiate the p53-dependent transactivation of pro-apoptotic genes, such as p53AIP1 [42], p21Waf1 [43], Noxa [44], Bax [45] and Puma [46], leading to cell death [7, 12]. p53 allows caspase-mediated cleavage of HIPK2 at D916/D977 (Figure 1). The resulting C-terminal truncated HIPK2 demonstrates an elevated p53 activity and cell apoptosis [19]. HIPK2 can also induce apoptosis through caspase-6 activation [47]. Intriguingly, more and more evidence supports the novel notion of HIPK2 being a key regulator of the NF-kB signaling pathway, which often promotes abnormal expression of tumor-associated genes that inhibits cell apoptosis and increases angiogenesis and tumor metastasis, thereby directly promoting the incidence and development of malignant tumors [48].

### HIPK2 decreases angiogenesis in a hypoxic environment

Strong evidence demonstrates that HIPK2 plays a role in hypoxic response *via* being a co-suppressor of hypoxia inducible factor 1α (HIF-1α), a major tumorigenic factor transactivating angiogenesis and invasion related genes [34].

Under hypoxia, repression of prolyl hydroxylation leads to the steady accumulation of HIF-1α. In turn, HIF-1α and HIF-1β dimerize to become the functional HIF-1 complex to increase the expression levels of cancer-promoting genes [49]. Thus, inhibiting HIF-1α activity may help enhance efficacy of conventional cancer therapy [50]. Hypoxia may down-regulate HIPK2 by inducing the p53 target MDM2 leading to both suppression of HIF-1α activity and p53-mediated apoptosis [51]. Nardinocchi et al. [13] used inhibition of HIF-1α by small inhibitory RNA (siRNA) to demonstrate that HIF-1α up-regulation induced proteasomal degradation of HIPK2. To sum up, hypoxia-induced ubiquitin ligases such as Seven in absentia 2 (Siah2), Seven in absentia 1 (Siah1) or MDM2, induce HIPK2 degradation which effectively inhibits therapy-induced p53 apoptotic activity, thereby promoting cancer progression [52–55].

Under hypoxia, HIPK2 is degraded in a proteasome-dependent and Siah1-dependent manner [56]. In normoxia, HIPK2 stability is maintained by a number of factors, with some HIPK2 proteins associated with Siah2. Hypoxic conditions commence marked increase in HIPK2/Siah2 association, resulting in rapid poly-ubiquitylation dependent proteasomal degradation of HIPK2. Under hypoxic conditions, Siah1 and Siah2 mediate the ubiquitination and proteasomal destruction of prolyl hydroxylase domain protein PHD1 and PHD3, which induces HIF-1α protein stabilization and increasing expression levels of HIF-1 target genes, e.g. vascular endothelial growth factor (VEGF) [57].

There are reports that WD40 domain and suppressor of cytokine signaling (SOCS) box protein-1 (WSB-1) is not only involved in sensing DNA damage by targeting HIPK2 for degradation, but that it is also a target of HIF-1 [58]. Hypoxia-induced HIPK2 degradation is reversed by WSB-1 loss. Inhibition of WSB-1 expression increases HIPK2 expression and promotes cell death in hypoxic cells [59]. In addition, HIPK2 silencing up-regulates HIF-1α and HIF-1 activity, resulting in increased VEGF levels, angiogenesis, and chemo-resistance [10, 60]. HIF-1α and VEGF up-regulation in HUVEC cells correlate with increased vascularity of *in vivo* xenografts and tube formation *in vitro* [10]. Increased vascularity following VEGF up-regulation directs tumor progression [61]. In brief, HIPK2 can inhibit angiogenesis by regulating VEGF, siah-1, siah-2, WSB-1 and HIF-1 in hypoxia.

### HIPK2 suppresses tumor invasion and metastasis

Tumor invasion and metastasis are complicated processes with multiple steps. Previous studies have confirmed that HIPK2 mediates tumor invasion and metastasis [9], alas with undefined mechanisms. HIPK2 activation induces Ser422 phosphorylation and degradation of CtBP to repress tumor metastasis [24]. Increasing body of evidence indicates that CtBP is a key promoter of carcinogenesis, especially in cancer metastasis [62]. CtBP participates in the down-regulation of E-cadherin gene, a marker of epithelial-mesenchymal transition (EMT) that contributes to cancer metastasis. Therefore, expression of CtBP is critically related to the malignant transformation of a number of cancers [63–65].

Studies have indicated that HIPK2 is involved in tumor invasion by inhibiting various signaling pathways [9]. Firstly, data have indicated that the well-studied wingless/int (Wnt) signaling pathway has a definite role in tumor invasion. Activation of this pathway can alter the expression of cell adhesion molecules, proteases and angiogenesis factors to promote cell invasion [66–68]. Secondly, knockdown of HIPK2 stabilizes β-catenin, increases nuclear localization of β-catenin, resulting in enhanced expression levels of Wnt target genes and cell proliferation *in vivo* and *in vitro*. HIPK2 down-regulates the *c-myb* proto-oncogene product by inhibiting the Wnt/β-catenin signaling pathway [69]. c-Myb is downstream effector of Wnt/β-catenin pathway, controlling a variety of developmental steps, inhibition of proliferation and invasion [70, 71]. Studies also have showed that that c-Myb protein is phosphorylated and degraded by Wnt1 *via* a pathway involving TGF- β -activated kinase (TAK1), HIPK2 and Nemo-like kinase (NLK) [72]. Wnt1 signaling activates the mitogen-activated protein (MAP), NLK and HIPK2. NLK induces phosphorylation of c-Myb at different sites by interacting with c-Myb and HIPK2, resulting in the ubiquitination and proteasome-dependent degradation of c-Myb. c-Myb regulates proliferation, differentiation and apoptosis *via* affecting transcription of target genes [73]. Meanwhile, Tan et al. showed that HIPK2 inhibition increased EMT and cell invasion, which was probably mediated by Wnt signaling [9].

**Figure 3:**
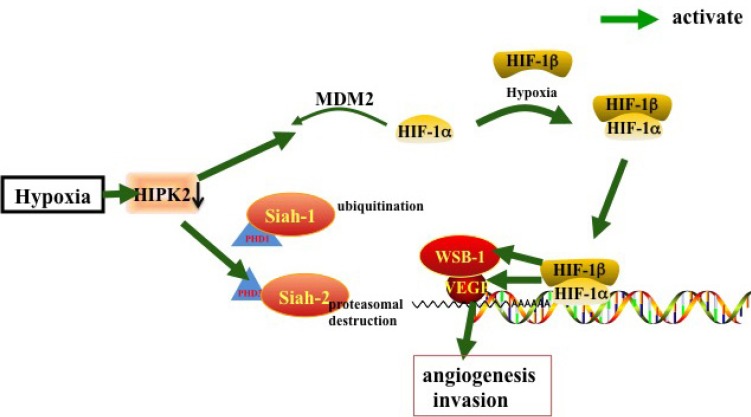
HIPK2 inhibits angiogenesis by regulating siah-1, siah-2, WSB-1, HIF-1 and VEGF in a hypoxic environment In hypoxia, HIF-1α dimerizes with HIF-1β to form the active HIF-1 complex, the PHD1/PHD3 mediated ubiquitination and proteasomal destruction of ubiquitin ligases Siah1 and Siah2 results in HIF-1α stabilization and activation of HIF-1 targeting genes such as VEGF. Hypoxia down-regulate HIPK2 to activate this pathway.

Thirdly, depletion of HIPK2 activates β4 transcription, leading to a significantly higher level of phosphorylation of β4-dependent mitogen-activated protein kinase (MAPK) and Akt, and consequent promotion of anchorage-independent growth and invasion [74]. HIPK2 knockdown also leads to HIF-1-mediated cyclooxygenase-2 (COX-2) up-regulation, which has been found to promote invasion in many types of cancer [75, 76, 77]. COX-2 expression is highly expressed in response to inflammatory mediators, growth factors, and oncogene activation, suggesting its association with cancer invasion and metastasis [78]. But whether HIPK2 directly down-regulates COX-2 to inhibit invasion and metastasis remains to be experimentally elucidated. Nodale et al. [79] found HIPK2-mediated vimentin down-regulation led to suppression of cancer cell invasion. In addition, evidence revealed that HIPK2 participated in the transforming growth factor beta (TGF-β)-JNK signaling dependent up-regulation of invasion and metastasis. RNA interference mediated HIPK2 knockdown inhibits TGF- β -induced JNK activation [8], and HIPK2 effect on JNK is modulated through dynamic SUMO-1 modification [80]. JNK signaling pathways are strongly associated with tumor progression and metastasis [81, 82]. Therefore, HIPK2 may decrease tumor invasion and metastasis through its regulation of JNK signaling pathways.

**Figure 4:**
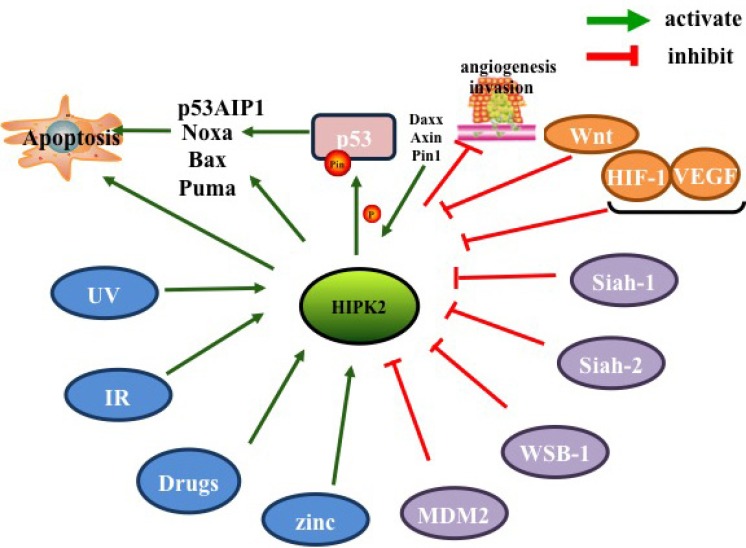
Factors inhibiting and promoting HIPK2

### HIPK2 attenuates multidrug resistance (MDR) in cancer

Multidrug resistance (MDR) represents one of a variety of mechanisms that cancer cells use to evade the cytotoxic effects of various anti-cancer drugs, thus decreases the efficacy of cancer therapeutics [83]. Inhibition of HIPK2 attenuates the adriamycin-induced apoptosis by decreasing pSer46-p53 levels, whilst HIPK2 overexpression induces apoptosis in chemoresistant cancer cells, along with induction of p53 Ser46-target gene AIP1 [84]. This conceptual result led to studies testing HIPK2 as a therapeutic target of gene therapy for chemoresistant ovarian cancer with wtp53 [12, 20, 84]. Lin et al. [85] found that HIPK2/Wip1 signaling might be a novel mechanism controlling chemoresistance. In detail, they confirmed that overexpression of HIPK2 sensitized chemoresistant cancer cells to cisplatin by inhibiting Wip1 expression. Hypoxia helps cancer cells to resist chemotherapy, whereas HIPK2 mediated repression of HIF-1α activity confers sensitization of chemoresistant cells with drug induced apoptosis [60]. Emerging data also indicate that HIPK2 silencing by siRNA impairs p53 tumor suppressor function, induces chemoresistance, and increases *in vivo* tumor growth [86]. HIPK2 inactivation unleashes signaling pathways that result in p53 loss-of-function and chemoresistance [12, 34]. Other observations showed that HIPK2 knockdown induced resistance to various anti-cancer drugs even by targeting ΔNp63α in p53-null cells [87]. These results suggest that HIPK2 can restore chemo sensitivity and inhibit chemo resistance.

### Conditions/factors that enhance HIPK2 activity

HIPK2 can be activated by numerous DNA damaging factors, including ultraviolet light (UV light), ionizing radiation (IR), genotoxic chemo-therapeutics [88], zinc in a hypoxic environment [13] and herbs used in traditional Chinese Medicine [89].

### Ionizing radiation (IR)

Studies have showed that IR induces the accumulation and activation of HIPK2.

IR-induced up-regulation of HIPK2 correlates with phosphorylation of p53 at Ser46, which is inhibited by RNAi mediated HIPK2 silencing. Interestingly, the DNA damage checkpoint ataxia telangiectasia mutated (ATM) kinase mediates IR induced HIPK2 activation [11] [90].

### Ultraviolet light

Upon sensing UV damage, HIPK2 is activated and causes Ser46 phosphorylates of p53, which next facilitates its acetylation at lysine 382, resulting in p53-dependent apoptosis [12]. After exposure to UV or cisplatin, HIPK2 and JNK1, provoke phosphorylation of CtBP at Ser422, to initiate apoptosis [91].

### Chemotherapy drugs

HIPK2 is activated by various anti-cancer drugs, including cisplatin (CDDP), adriamycin (ADR) and roscovitin, to form HIPK2/p53Ser46 apoptotic signaling pathway. Therefore, HIPK2 is the key factor of p53 activity in response to chemotherapeutic drugs [92]. In chemoresistance, deregulation of HIPK2/p53 Ser46 signaling occurs. For conceptual novelty, exogenous HIPK2 should offer a valuable and promising new treatment option to circumvent inhibition of apoptosis for women with chemo-resistant ovarian cancer [84].

### Zinc

In addition to DNA damaging agents, HIPK2 can be activated by zinc in hypoxia and in chemoresistance. Zinc restored protein levels of HIPK2 and the chemo-sensitivity in cancer cells [13]. Zinc up-regulated HIPK2 to inhibit COX-2 level, leading to reduced cancer growth [75]. Some study even showed that zinc could reactivate HIPK2 resulting in HIF-1 pathway suppression, thereby restoring p53 apoptotic activity [93]. p53 protein misfolding in HIPK2 knockdown context was reverted by zinc supplementation [94]. Therefore, zinc treatment should be used in combination with other anticancer therapeutics to restore the HIPK2/p53 apoptotic signaling pathway.

### Herbs used in traditional chinese medicine

The reason Traditional Chinese medicine comes to our attention in this review is because of a study revealing inhibition of HIF-1 and S100A4 by saponin extracts of ginsenoside (Ginsen) and Gynostemma, as well as coix polysaccharides, resulting in the suppression of cancer cell migration and invasion [95]. Verbascoside (VB), extracted from a Traditional Chinese medical plant genus, effectively activates HIPK2/p53 signaling pathway in human colorectal cancer (CRC), resulting in increased CRC cell apoptosis [89].

## CONCLUSIONS

HIPK2 is a multi-functional signaling molecule and a tumor suppressor that mediates growth, regulation and apoptotic cellular responses. HIPK2 induces cell death by activating p53-dependent [7, 22, 31, 32] and independent pathways (including the JNK signaling pathway) [8, 80], to promote tumor cell apoptosis. In a hypoxic environment, HIPK2 down-regulates the activity of HIF-1 [34], Siah1 [56], Siah2 [3], VEGF [57] and WBS-1 [59] to inhibit tumor angiogenesis [50]. Additionally, several studies have shown that HIPK2 can also decrease tumor cell invasion and metastasis by Wnt/β-catenin [69], CTBP [24], JNK [8, 80–82] and COX-2 signaling pathways [78]. HIPK2 can adjust MDR to increases sensitivity of cells to chemotherapy drugs [12, 20, 84]. HIPK2 can also be activated by DNA damage (i.e. ionizing radiation, UV light) [88], anti-tumor drugs (i.e. cisplatin, adriamycin, roscovitine) [92], zinc in a hypoxic environment [13], and Traditional Chinese medicine [89]. In summary, HIPK2 inhibits cancer cell tumorigenesis, and promotes pro-apoptotic gene expression. HIPK2 can serve as a novel biomarker in tumors as well as a potential target for anti-cancer therapies.
